# Acute cerebellitis as a post-infectious complication of primary varicella in an immunocompetent young adult: A case report

**DOI:** 10.1016/j.idcr.2026.e02805

**Published:** 2026-10-17

**Authors:** Alireza Davoudi Badabi, Shadi Shayestehazar, Bahar Najafi, Farzaneh Mohammadzadeh Rostami

**Affiliations:** a Antimicrobial Resistance Research Center, Communicable Diseases Institute, Mazandaran University of Medical Sciences, Sari, Iran; b Department of infectious disease, School of Medicine, Mazandaran University of Medical Sciences, Sari, Iran

**Keywords:** Varicella-zoster virus, Cerebellitis, Truncal ataxia, Adult, Varicella, Case report

## Abstract

**Background:**

Acute cerebellitis is a well-documented neurological sequela of primary varicella-zoster virus (VZV) infection. Although frequently reported in children, this complication remains uncommon in adults and may present diagnostic difficulties, especially when neuroimaging studies are normal.

**Case Presentation:**

A previously healthy, immunocompetent 20-year-old man was admitted with a four-day history of progressive gait instability and imbalance. Nine days prior to admission, he had developed a generalized vesicular rash consistent with primary varicella, initially involving the trunk and subsequently spreading to the face and extremities. He also reported occipital and frontal headaches, undocumented fever during the active phase of the rash, and postprandial nausea with vomiting. Neurological examination revealed intact consciousness, normal cranial nerve function, truncal ataxia, a wide-based gait, impaired tandem gait, and inability to maintain balance during Romberg testing even with eyes open. No appendicular ataxia was detected. Laboratory studies showed a normal complete blood count and an erythrocyte sedimentation rate of 13 mm/hour. Brain computed tomography and magnetic resonance imaging revealed no acute pathological findings. Cerebrospinal fluid polymerase chain reaction (PCR) was positive for VZV. Intravenous acyclovir (750 mg three times daily) was initiated, with subsequent gradual clinical improvement.

**Conclusion:**

Post-varicella cerebellitis should be included in the differential diagnosis of adults presenting with acute truncal ataxia following recent primary varicella infection, even when neuroimaging is normal. Cerebrospinal fluid VZV PCR is a valuable diagnostic aid, and early antiviral therapy may be associated with favorable outcomes.

## Introduction

Varicella-zoster virus (VZV), a neurotropic alphaherpesvirus, is the etiological agent responsible for primary varicella infection (chickenpox). Although primary varicella typically follows a benign, self-limited course in immunocompetent children, it may be associated with a diverse spectrum of neurological complications, including encephalitis, meningitis, vasculopathy, myelitis, and cerebellitis [1]. Among these, acute cerebellitis ranks among the most frequently documented neurological manifestations in the pediatric population; however, its occurrence in adults remains comparatively uncommon [1], [2] The classic clinical presentation of post-varicella cerebellitis encompasses gait disturbance, truncal or appendicular ataxia, dysarthria, nystagmus, and headache, typically manifesting between several days and three weeks following the onset of the characteristic vesicular rash [3], [4]. Neuroimaging findings are heterogeneous, ranging from entirely normal studies to diffuse cerebellar edema or overt inflammation. Given the rarity of this condition in adults and the potential for unremarkable neuroimaging, the diagnosis frequently necessitates a high index of clinical suspicion, supported by targeted virological confirmation, such as polymerase chain reaction (PCR) of the cerebrospinal fluid (CSF) [4].

Herein, we describe a case of acute cerebellitis following primary varicella infection in an immunocompetent young adult who presented with progressive truncal ataxia and demonstrated a positive CSF VZV PCR, despite entirely unremarkable brain imaging.

## Case presentation

A 20-year-old immunocompetent man with no significant past medical history was admitted due to progressive imbalance and gait disturbance, which had begun four days prior to presentation.

Nine days before admission, he had developed a generalized vesicular rash suggestive of primary varicella infection. The lesions initially appeared on the trunk and subsequently spread to the face and extremities. At the time of admission, crusted lesions and post-inflammatory hyperpigmentation were visible throughout the patient's body. During the active phase of the rash, he reported episodes of undocumented fever. Four days prior to admission, he developed progressive gait instability accompanied by occipital and frontal headaches. He also experienced postprandial nausea and vomiting, particularly while sitting or standing. There was no history of altered mental status, behavioral changes, seizures, focal weakness, sensory deficits, visual disturbances, or loss of consciousness.

Upon admission, vital signs were stable. The patient was fully alert and oriented. Cranial nerve examination was unremarkable. Neurological assessment demonstrated marked truncal ataxia, a wide-based gait, impaired tandem walking, and an inability to maintain balance during Romberg testing, even with eyes open. Limb coordination tests revealed no evidence of appendicular ataxia. No motor or sensory deficits were identified. Laboratory evaluation showed a normal complete blood count without leukocytosis and an erythrocyte sedimentation rate of 13 mm/hour. Brain computed tomography and magnetic resonance imaging revealed no acute pathological abnormalities.

CSF analysis returned a positive PCR for varicella-zoster virus, supporting the diagnosis of post-VZV cerebellitis. The patient was treated with intravenous acyclovir at a dose of 750 mg three times daily. After one week of therapy, gradual improvement in gait stability and neurological symptoms was observed. Following one week of hospitalization, the patient was discharged in stable condition with partial resolution of symptoms.

## Discussion

Acute cerebellitis is a well-recognized neurological complication following primary varicella-zoster virus (VZV) infection [5]. While the majority of cases occur in the pediatric population, acute cerebellitis in adults is rare and can present a significant diagnostic challenge due to the broad differential diagnosis of acute-onset ataxia in this age group, including stroke, paraneoplastic syndromes, multiple sclerosis, and toxic-metabolic causes [6]. In our patient, the clear temporal relationship between the onset of the characteristic varicella rash and the development of cerebellar symptoms aligns well with previously reported cases. Neurological manifestations of post-varicella cerebellitis typically emerge within days to several weeks after the rash appears [3]. The predominant finding was truncal ataxia with gait impairment, without significant limb ataxia or dysmetria. This pattern strongly suggests preferential involvement of the cerebellar vermis, which plays a critical role in axial balance and postural control [7]. Neuroimaging findings in VZV-associated cerebellitis are highly variable. While some patients show cerebellar signal hyperintensities or edema on MRI, normal CT and MRI scans—as observed in our patient—do not rule out the diagnosis [8]. This case underscores the importance of integrating clinical history, neurological examination, and laboratory data, particularly cerebrospinal fluid (CSF) analysis, when evaluating suspected cerebellitis. The positive CSF VZV PCR provided definitive evidence of central nervous system involvement and is now considered a cornerstone in the diagnosis of VZV-related neurological disease [9]. Uncomplicated VZV cerebellitis is generally a self-limited condition that resolves within 2–4 weeks, irrespective of antiviral therapy [10]. In more severe cases with cerebellar edema and raised intracranial pressure, glucocorticoid therapy and occasionally decompressive interventions may be necessary [11]. Our patient was treated with a 6-day course of intravenous acyclovir. Following one week of hospitalization, the patient demonstrated gradual but steady clinical improvement and was discharged without residual neurological deficits.

This case highlights that VZV cerebellitis should be considered in the differential diagnosis of acute ataxia in adults, even in the absence of typical skin lesions at the time of neurological presentation or with normal neuroimaging. Early recognition and prompt antiviral treatment may contribute to favorable outcomes. Further studies are needed to better define the optimal duration of antiviral therapy and the role of adjunctive corticosteroids in adult patients (Fig. 1, Fig. 2).

**Fig. 1:**
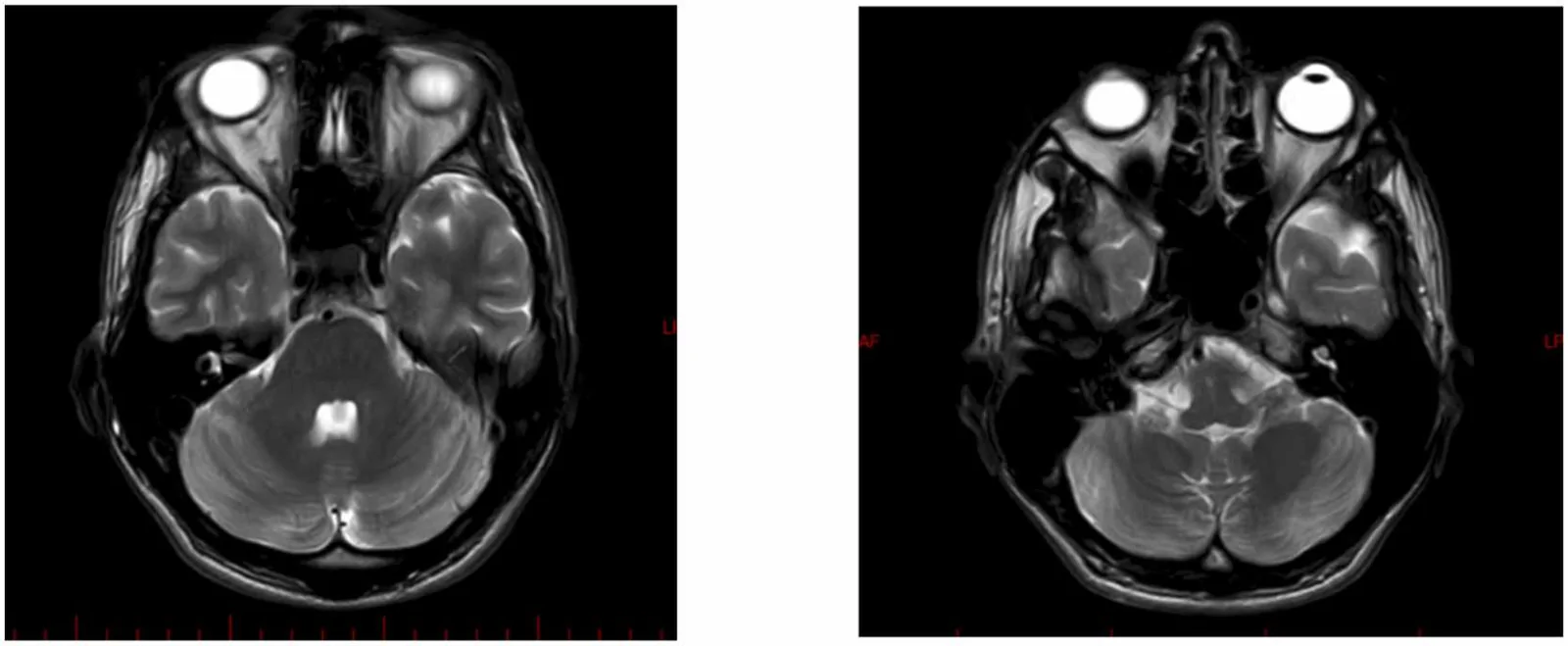
T2 axial brain MRI demonstrating no abnormality in the cerebellum.

**Fig. 2:**
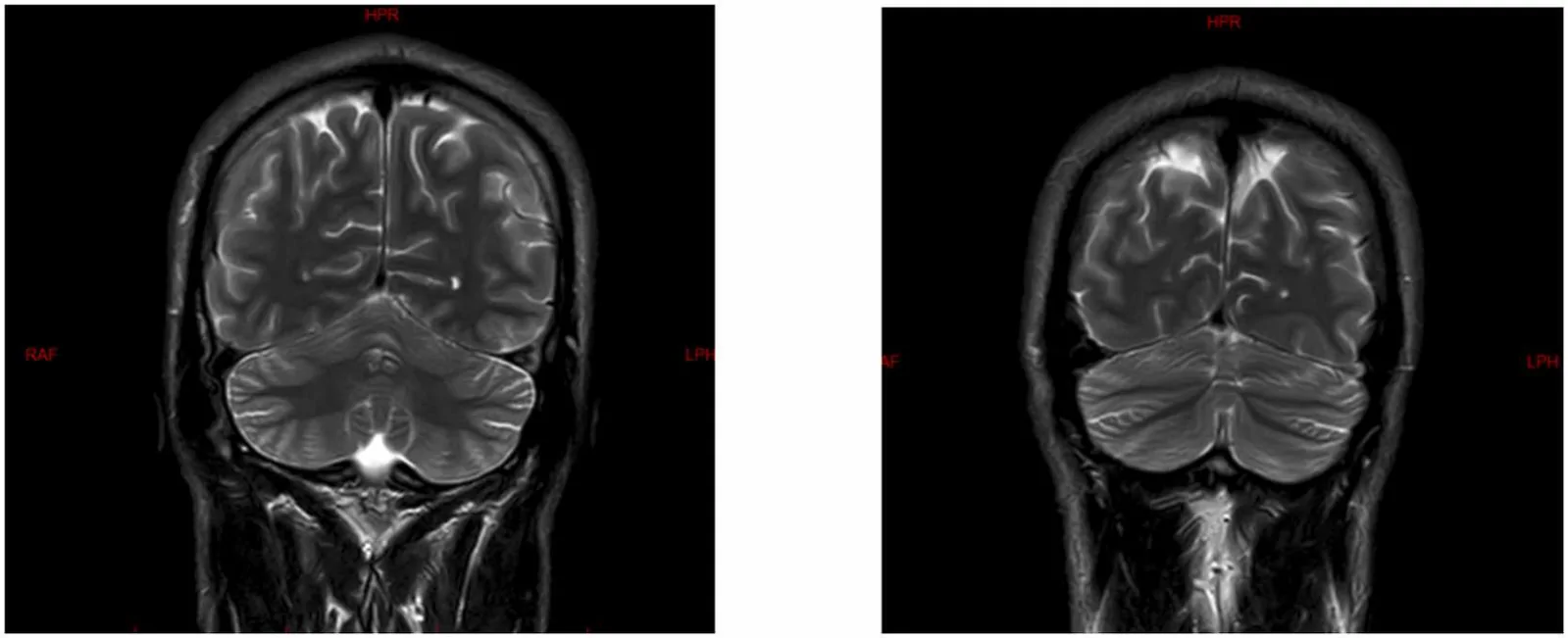
T2 coronal brain MRI demonstrating no abnormality in the cerebellum.

## Conclusion

This report highlights that acute cerebellitis should be included in the differential diagnosis of acute ataxia in immunocompetent adults, even in the setting of normal brain imaging. Detection of VZV DNA in cerebrospinal fluid by PCR provides rapid and reliable confirmation of the etiology. Prompt antiviral treatment appears to be associated with excellent neurological recovery in uncomplicated post-varicella cerebellitis. Increased awareness of this entity is essential for timely diagnosis and optimal management in adult patients.

### Funding

The study was funded by 10.13039/501100004160Mazandaran University of Medical Sciences (Grant/Tracking ID: 26001; Ethics Code: IR.MAZUMS.REC.1405.138).

## CRediT authorship contribution statement

**Alireza Davoudi Badabi:** Validation, Supervision, Funding acquisition, Conceptualization. **Shadi Shayestehazar:** Visualization, Validation, Project administration, Data curation, Conceptualization. **Bahar Najafi:** Writing – review & editing, Writing – original draft, Investigation, Data curation. **Farzaneh Mohammadzadeh Rostami:** Writing – review & editing, Writing – original draft, Investigation, Funding acquisition.

## Ethical approval

This study was conducted in accordance with the Declaration of Helsinki. The study protocol was reviewed and approved by the Ethics Committee of Mazandaran University of Medical Sciences (Ethics code: IR.MAZUMS.REC.1405.138). Written informed consent was obtained from the patient for publication of this case report and any accompanying images. All patient data were anonymized to ensure confidentiality.

## Declaration of Competing Interest

The authors declare that they have no known competing financial interests or personal relationships that could have appeared to influence the work reported in this paper.
